# Worsening alopecia areata revealing immune thrombocytopenic purpura and Graves disease in a child: Divergent manifestations in monozygotic twins

**DOI:** 10.1016/j.jdcr.2026.09.046

**Published:** 2026-10-01

**Authors:** Kijae Sung, Kyung-Hwa Nam, Jin Park

**Affiliations:** a Department of Dermatology, College of Medicine, Jeonbuk National University, Jeonju, Korea; b Research Institute of Clinical Medicine of Jeonbuk National University-Biomedical Research Institute of Jeonbuk National University Hospital, Jeonju, Korea

**Keywords:** alopecia areata, Graves’ disease, immune thrombocytopenic purpura, monozygotic twins, multiple autoimmune syndrome, polyautoimmunity

## Introduction

Alopecia areata (AA) is an immune-mediated disorder that is frequently associated with other autoimmune conditions.^1^ While its association with autoimmune thyroid disease is well established, co-occurrence with immune thrombocytopenic purpura (ITP) has rarely been reported, despite both conditions being immune-mediated. We report a pediatric patient with AA in whom further evaluation revealed concurrent ITP and Graves’ disease, highlighting divergent autoimmune manifestations between monozygotic twins with shared AA and Graves’ disease.

## Case report

A 13-year-old girl with a history of AA presented with worsening hair loss. At presentation, she exhibited extensive nonscarring alopecia across the scalp, with a Severity of Alopecia Tool score of 69.9%, representing a marked increase compared with her prior disease course, which had consisted of limited patches that regrew after intralesional corticosteroid treatment (Fig 1 and Supplementary Table I, available via Mendeley at https://doi.org/10.17632/rc55dn6665.1). Before thrombocytopenia was detected, the current episode had shown insufficient clinical response to intravenous methylprednisolone pulse therapy, oral cyclosporine, and topical treatment.

**Fig 1:**
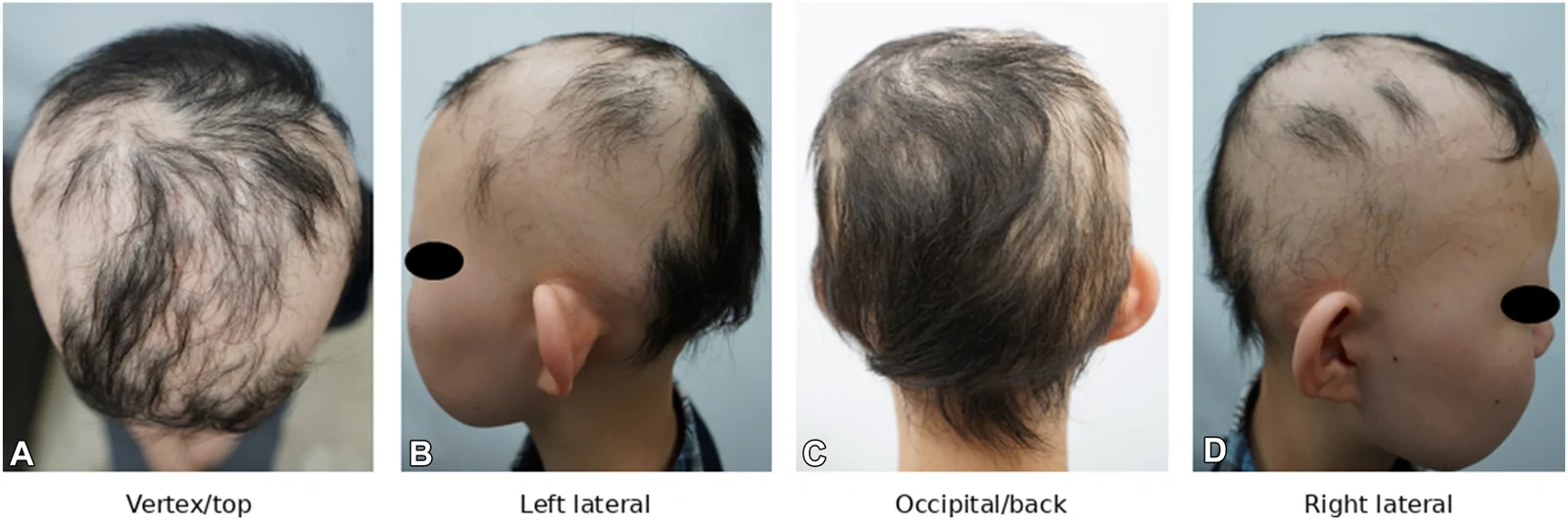
Clinical presentation of alopecia areata. **A,** Vertex/top, **(B)** left lateral, **(C)** occipital/back, and **(D)** right lateral views show extensive nonscarring alopecia across the scalp at presentation, with a Severity of Alopecia Tool score of 69.9%.

Given the rapid progression, extensive involvement, and poor response to treatment, laboratory studies were obtained to reassess for systemic comorbidities and to evaluate suitability for alternative systemic therapy. These studies revealed marked thrombocytopenia (platelet count, 27,000/μL) with otherwise normal coagulation studies. Subsequent directed history and examination disclosed a 1-month history of easy bruising and purpuric changes on the anterior neck that had not previously been reported. No evidence of infection, nutritional deficiency, or hemolysis was identified on additional testing. Bone marrow evaluation and an inherited bone marrow failure syndrome gene panel did not suggest an alternative underlying etiology.

Concurrent thyroid evaluation revealed suppressed thyroid-stimulating hormone levels (<0.005 μIU/mL), elevated free thyroxine (48.70 pmol/L), elevated triiodothyronine levels (2.15 ng/mL), and increased thyroid-stimulating immunoglobulin (7.6 IU/L). Thyroid ultrasonography and radionuclide imaging findings were compatible with Graves’ disease.

During follow-up, Graves’ disease was relatively well controlled with methimazole. In contrast, thrombocytopenia worsened to a nadir of 6 × 10^3^/μL and was treated with intravenous immunoglobulin and systemic corticosteroids, followed by deflazacort tapering. The platelet count improved b ut did not normalize, reaching 78 × 10^3^/μL at the most recent assessment (Supplementary Fig 1 and Table I, available via Mendeley at https://doi.org/10.17632/rc55dn6665.1). Apart from AA in her monozygotic twin sister, no other family history of autoimmune disease was reported. In light of the patient’s diagnoses, further evaluation of her twin sister was undertaken. Her AA course was milder and more treatment-responsive than that of the patient, with documented Severity of Alopecia Tool scores ranging from 9% to 35% during follow-up and repeated hair regrowth observed during topical immunotherapy with diphenylcyclopropenone. Although she had no thyroid-related symptoms, evaluation prompted by the patient’s diagnoses revealed Graves’ disease (thyroid-stimulating hormone, 0.008 μIU/mL; free thyroxine, 34.20 pmol/L; thyroid-stimulating immunoglobulin, 2.8 IU/L), whereas serial platelet counts remained within the reference range (Supplementary Table II, available via Mendeley at https://doi.org/10.17632/rc55dn6665.1).

## Discussion

This case illustrates a rare concurrence of AA, ITP, and Graves disease in a pediatric patient. While AA is commonly associated with autoimmune thyroid disease, its coexistence with ITP has been reported only in a limited number of cases.^2^, ^3^, ^4^

In our patient, systemic comorbidities were identified when AA showed a marked change in behavior, with rapid progression and treatment refractoriness. This observation suggests that alterations in disease pattern may serve as a clinical clue to underlying systemic involvement. It is possible that the increased severity and treatment resistance of AA in this patient reflect broader systemic immune dysregulation, rather than a process confined to the hair follicle.

Previous reports have described synchronous improvement or deterioration of AA and ITP.^5^, ^6^ A recent case report demonstrated concurrent improvement of both conditions with baricitinib.^7^ These findings support the possibility of shared immunologic pathways, particularly involving T-cell-mediated responses and cytokine signaling. Although Janus kinase inhibition was considered in light of reports describing concurrent improvement of AA and ITP, it was not pursued because thrombocytopenia was already under active hematologic management and showed a partial but incomplete response to corticosteroid-based therapy. Given the limited case-based evidence supporting Janus kinase inhibitors for ITP, continued hematologic treatment with close monitoring was favored.

The findings in the monozygotic twin provide additional insight. The co-occurrence of AA and Graves’ disease in both sisters suggests a shared predisposition to autoimmunity. However, the absence of ITP in the twin sister to date and the differences in AA treatment responsiveness indicate that shared genetic background alone does not determine disease expression. Because future autoimmune manifestations in the twin sister cannot be excluded, and because human leukocyte antigen typing or polyautoimmunity-focused genetic analysis was not performed, the relative contributions of genetic susceptibility and environmental or stochastic immune triggers remain uncertain.

Both sisters meet the definition of polyautoimmunity, whereas the patient, with 3 autoimmune diseases, fulfills the definition of multiple autoimmune syndrome (MAS).^8^, ^9^, ^10^ Although the combination of AA, ITP, and Graves disease does not fit neatly within the traditional MAS subtypes, it remains consistent with the contemporary concept of MAS as part of the broader spectrum of polyautoimmunity.^8^, ^10^

Overall, this case highlights that rapid progression or unexpected treatment refractoriness in patients with AA may provide an opportunity to reassess for clinically occult comorbidities. The pattern of shared and divergent autoimmune manifestations in these monozygotic twins further suggests that genetic susceptibility may contribute to autoimmune clustering without fully determining individual disease expression.

### Declaration of generative AI and AI-assisted technologies in the writing process

None.

## Conflicts of interest

None disclosed.
